# Cost transferability problems in economic evaluation as a framework for an European health care and social costs database

**DOI:** 10.1186/s12962-021-00294-4

**Published:** 2021-07-18

**Authors:** Leticia García-Mochón, Joan Rovira Forns, Jaime Espin

**Affiliations:** 1 Andalusian School of Public Health, Cuesta del Observatorio 4, 18011 Granada, Spain; 2 CIBER en Epidemiología y Salud Pública (CIBERESP), Spain/CIBER of Epidemiology and Public Health (CIBERESP), Madrid, Spain; 3 Instituto de Investigación Biosanitaria ibs, Granada, Spain

**Keywords:** Transferability, HC cost database, Economic evaluation, Health technology

## Abstract

This article presents part of the work within Work Package 3 (WP3) of Impact HTA (Improved methods and actionable tools for enhancing HTA), a H2020 EU-funded research project, intended to enhance and promote collaboration in HTA across EU MS. Amongst other objectives, and in close collaboration with WP4, WP3 addressed setting up a multi-country unit-cost database: the European health care and social costs database (EU HCSCD). The purpose of the database is to facilitate the transference of healthcare economic evaluation analyses across countries, jurisdictions and settings. WP3 concentrates on healthcare costs; WP4 on social costs. This paper discusses the state of the art on this topic, building an appropriate conceptual and theoretical framework for Database development. We conducted a broad, but not systematic, literature and gray-literature review (LR), identifying existing practices and problems, and their implications, described in the Results section. We discuss practical implications and draw important conclusions behind the construction, and future evolution, of this database.

**Supplementary Information:**

The online version contains supplementary material available at 10.1186/s12962-021-00294-4.

## Background

Health Economic Evaluation (HEE) is a component of Health Technology Assessment (HTA) increasingly demanded by health sector decision-makers to inform priority setting towards efficient resource allocation. But economic evaluation analyses are time-consuming exercises that require specialized technical staff, skills which may not be available in some settings, or be too scarce in relation to the amount of analyses required [1]. Moreover, HEE analyses should be regularly updated and revised, as most of the variables that define cost-effectiveness ratios change over time: new treatments and technologies appear, input prices vary, etc. A decision-maker, under time and/or budget constraints, might not be able to generate de-novo analyses, of sufficient quality, to inform a relevant decision, on time, within a specific site and context, and might instead rely on existing similar studies, carried out somewhere else. This approach could be seen as more efficient use of expert capacity: wider availability of HEE results across sites would reduce the cost of carrying out individual analysis from scratch in each jurisdiction [2]. However, a straightforward re-utilisation of previous analyses may be neither valid, nor legitimate. This often-discussed problem of generalizability—similar to that of internal vs. external validity in experimental studies—could expose local decisions to important biases. HEE analyses, like all forms of HTA, must be contextualised, adjusted, taking into account—amongst other factors—key local conditions of the country, jurisdiction or setting where the technology is to be applied. In particular, resource utilization and monetary values differ substantially across countries and settings, probably as much, or even more, than the health outcomes of treatments. While results of an original HEE analyses might not be directly generalisable to other contexts, parts of it—e.g., the core clinical/epidemiological model—might still be of fair value, and could be applied o transferred to new or different setting.

Transferability of HEE is built on the assumption that at least some components of the original analysis or model can be generalisable and hence reusable in other settings [3]. For instance, it is often assumed that evidence of efficacy derived from clinical trials is not applicable (generalisable) to effectiveness, to effects in actual practice (external validity) [4]. However, it is usually assumed that the same evidence of efficacy carried out in a certain jurisdiction are, in principle, valid for populations in other settings. This is indeed the implicit assumption of many national Drug Regulatory Agencies when they grant market authorisation in a given country to new technologies on the basis of clinical trials carried out elsewhere.

The core component of a HEE analysis is a mathematical model or algorithm that represents the expected course of a disease under two or more health interventions. It seems therefore acceptable that the health outcomes and sometimes also the health resource utilisation of the interventions assessed might be validly projected in different settings by a single (the same) epidemiological-clinical model. But these assumptions should not be taken for granted, and the models and, ideally, underlying assumptions should always be validated.

As far as costs are concerned, transferability requires separately considering units of resources and unit monetary values (price or cost) of the resource, a standard recommendation and practice in HEE [4]. But such units would be equivalent, across two or more settings, only if medical resources, technologies and practices are the same in all settings. New technologies are likely to respond to similar, standardised protocols across EU countries, and use equivalent units. However, in a HEE analysis, their relevant comparators are far less likely to be uniform across the same countries, strongly reducing transferability [5]. Moreover, units of resources used across settings, might also differ. For instance, in the original study country, a comparator (usual care) may consist in one Primary Health Centres (PHC) visit, while in the target country it may mean three visits. Therefore, number of resource units is something that must be customized to the target setting, when transferring an EE study.

Even when resource use between study settings is equivalent, the likelihood that unit values of health care resources are also equivalent or highly similar between the same settings is low. In that situation—equal enough health intervention processes between countries—the unit costs/prices of the original study should be replaced by the respective unit costs/prices in the local setting where the core of the original study is to be applied [4].

The specific objective of this article is to review the problem of transferability in health economic evaluation, focusing on resource use and cost issues, with the final aim of providing an appropriate conceptual framework for the design and development of the European health care and social costs database.

## Methodology

We conducted a narrative literature review to identify studies of transferability in economic evaluation of health technologies, focusing on debate around transferability of resource use and cost. The search was conducted in February 2020 in MedLine and Web of science database and Google scholar. In addition, selected sources of gray literature were searched. To execute the review, the following terms were used: “transferability”, “generalizability”, combined with terms about health care cost and economic evaluation (“economic evaluation” “cost-effectiveness analysis”,” cost–benefit analysis” “health care cost” “cost analysis”) and other terms about tools, cost databases and guidelines (“tools”, “toolkit”, “cost database”, “standard cost list” “guideline”, check lists”). The web appendix (Additional file 1: Table S1) provides details of the bibliographic terms used and the search results obtained from Medline and Web of Science databases. Additionally, citation tracking in Google Scholar was used as well as a manual search of the reference lists of included studies. The search was restricted to the last 15 years and for studies published in Spanish and English.

Inclusion criteria includes studies that specifically addressed the cost transferability in the economic evaluation of health technologies, and studies or papers about cost database or standard cost lists. The exclusion criteria were: articles, for which full text was not available, were not in English or Spanish. From the articles retrieved in the first round of search, additional references were identified by a manual search among the cited references.

As the topic of our study, and the consequent LR, was quite broad, we grouped findings in the five sections:

1. How is transferability defined in the literature?
2. Tools and method to assess the transferability in Economic Evaluations.
3. Critical factors for transferring cost in Economic Evaluation of health technologies.
4. What do national economic evaluation guidelines say about cost transferability?
5. Health care unit cost database and standard cost list for health care.
6. Usefulness of an European health care and social costs database.

## Results

### How is transferability defined in the literature?

In our review, we found confusion around some terms that refer to the (re)utilization of an empirical study or analysis in a different setting or context of the original one where the study was carried out or where the results were intended to apply. In clinical research, there is a clear distinction between “internal validity” and “external validity”. The efficacy of a technology or intervention refers to the effects on a target variable, measured in an experimental setting, whereas effectiveness refers to the effects of the same technology or intervention in real medical practice. Efficacy is generally assumed to be larger than effectiveness because the former takes place in a controlled, ideal environment, under conditions highly favorable for the technology to achieve the intended effects.

However, in actual practice patients can seldom be selected to match all experiment criteria. For instance, they might not be equally well diagnosed, investigated and followed, adherence and compliance tends to be lower, and hidden variables might introduce new sources of bias. Manuals and Guidelines for HEE recommend efficacy data from clinical trials to be appropriately adjusted to real practice conditions, in order to not overvalue an intervention’s effectiveness, which is what the decision maker assigning resources needs to ascertain [6].

A similar problem arises when a HEE study intended for a given setting or jurisdiction is used at a different setting or jurisdiction. Most authors frame this as “transferability” and/or a “generalizability” problems, using both terms as synonymous, related to external validity [7–9] but others, assign different meaning to these terms. For instance, according to Walker [10], transferability is “the ability to extrapolate results obtained from one setting or context to another”, differentiating between the potential (or generic) transferability of a study and its actual (or specific) transferability to another policy or practice. Potential transferability hinges on how fully the intervention has been described, how comprehensively the implementation context is described and which patient or participant groups were selected for exposure to the intervention. This allows practitioners or policy makers elsewhere to compare variables against their own options, target populations or organisational contexts. Of note, this type of transferability is a property of a particular original study, including what it has evaluated and how fully it has described issues. In contrast, actual transferability assesses the same phenomena described above, but in relation to a particular decision or policy choice in a particular jurisdiction, population and health system. Actual transferability is a property of the original program, study and setting, and the population, setting and potential constraints on program design and funding, in the place where the same program may be applied. Therefore, it is not a property of an individual study or program, but changes depending on where and when original evidence is to be transferred to.

On the other hand, generalizability of results is defined as being ‘similar to external validity in that it refers to the extent to which information (both clinical and economic) can be extrapolated to either a patient group with different characteristics or to a similar patient group treated in a different geographic, political or time structure’. A similar position is held by Schulper et al. [5], who defines generalizability as ‘the degree to which the results of an observation hold true in other settings’. In the clinical evaluation literature, issues of generalizability focus mainly on the characteristics of patients in a given study and how representative they are of a broader population.

Boulenguer et al. [8], proposes clarification in the terms transferability and generability by considering at least two ways in which economic evaluations can be used by decision makers in different settings: (a) by applying the conclusions directly because the results are either assumed or assessed to be relevant to the new setting (for example, assuming that since the use of drug A for disease B has proven to be cost-effective in country C, it will also be a cost-effective treatment in country D) and (b) by stating that a given study is transferable if (a) potential user(s) can assess their applicability to their setting and they are applicable to that setting.

Therefore, transferability is a broader concept than generalizability. If researchers desire to make the results of their studies transferable to other contexts, they must keep a detailed account of salient points surrounding their research, including a sufficiently rich description of their methods.

Barbieri et al. [3] states that studies may be considered generalizable if they can be applied to a range of jurisdictions without any adjustment needed for interpretation. Some studies may be transferable if they can be adapted to other settings, while other may be so specific to a given jurisdiction that they are simply not transferable to any other jurisdiction. This is almost identical to the Task Force’s working definitions: generalizability, applying the results of a study to a number of countries without needing to adjust for interpretation, and transferability, adapting the results of a study to other countries) are other challenges that have been identified in the literature [11].

For the purposes of our analysis and the usefulness of the European health care and social costs database (EU HCSCD), we will share the definitions of generalizability and transferability by Drummond [11] and by Barbieri et al. [3].

It might be assumed that generalizability of an EE study -applying the results to a number of countries without needing to adjust for differences- is only valid in very few instances, i.e. is limited to countries and jurisdictions that are very similar—to the original study country—in all characteristics/variables that determine the results of the study (costs, effectiveness, ICER, etc.).

There are also problems of scale. The term “countries” can be too narrow and misleading; problems of transferability and generalizability may apply in smaller areas. In this report “setting”, “jurisdiction” and “country” are often used interchangeably, because they share transferability problems.

The EU HCCD includes unit costs that mainly refer to countries/nations, but might be extended to costs representing regions or smaller areas and settings, if they are relevant jurisdictions for decision making and unit cost data are available and can be collected. If there are within-country regions or jurisdictions characterized by large differences in costs, assessing the cost-effectiveness of a decision should ideally be based on the differential costs at each region/jurisdiction/setting. That is, an intervention that reduces length of hospital stay after a surgical procedure, might be cost effective for a tertiary hospital with a high cost per day but not cost-effective for a local hospital with much lower hospitalization costs. But, although locally-contextualized analysis is the best approach for a local decision maker considering reimbursement of a given technology, a decision making body sitting at national level might have to use average conditions for calculating an ICER for making P&R (pricing and reimbursement) decisions at central level.

In fact, Canada, one of the first countries to regularly apply EE analysis to P&R decisions in health technology, enforced province-specific pharmacoeconomic methodological guidelines and unit costs lists, e.g. for Ontario, Alberta, etc. [12, 13].

Although different settings might have highly similar characteristics that allow a HEE study done in one of them to be valid and hence applicable to the others “without needing to adjust for interpretation”, this is a rather unlikely situation. In most cases, analyses will need to be adjusted or adapted in order to customize or properly contextualize the results of an original study to a different country.

In order to make the process of adaptation feasible, the original analysis should be totally transparent and reproducible. For this, the analyst considering transferability would be able to identify key parameters and assumptions, and assess whether they apply to the target country conditions. It is important to mention that high similarity between jurisdictions refers not only to objective variables, such as the characteristics of the population and the health system, or the health technologies applied—especially, the potential comparators—but also to more intangible factors, such as any methodological guidelines in force in the target jurisdiction.

Healthcare costs are often calculated as number of units of the resource times the unit cost. Regarding unit quantity of each resource, the analyst must first ensure that the figures registered in the original EE study for all cost components, including, for instance, the number of units of each disposable goods used (lab tests, medicines, and other consumables), the time spent by all categories of health care personnel, the time use of fixed equipment, etc. are either generalizable to the target setting, or have been appropriately adjusted to match the conditions in the target country. The analyst should then focus on appropriate and valid unit costs of the resources in the target country. This is likely to be tedious and time-consuming, even unfeasible, because such data might not be available at all or be difficult to locate and validate.

Once local (target country) unit costs are identified, in order to ensure the validity of the transference of the unit cost, it is essential that the resources, i.e., the monetary values to be interchanged or substituted, are precisely defined and described in detail in both settings. For instance, if the unit cost to transfer is a PHC doctor’s first visit, the assessor should verify that this includes the same components in either setting and that costing and accounting procedures are equivalent. These caveats are seldom explained in sufficient detail in EE reports and, consequently, unit cost adaptation carried out when transferring EE analyses becomes a “black box” whose validity cannot be properly assessed.

### Tools and method to assess the transferability in economic evaluations

Several tools have been developed to assist with the challenges of adapting studies or data from other jurisdictions. Some of them are checklists for evaluating the generalizability of economic evaluation, such as the checklist from Drumond [4], Boulenguer [8], Turner [14], and Nixon [15]. Other studies propose a sequenced flow-chart-type approach to help decide if a study can be validly transferred (for instance, Welte’s transferability decision chart (2004), Drummond’s application algorithm [11]). Other proposals refer to check-lists that are summarized as Heyland’s generalizability criteria indexes [16], Späth’s transferability indicators, 1999 and Antonanzas’ transferability index 2009 [17]. This topic has been recently reviewed by Goeree et al. [9].

Moreover, the European Network for Health Technology Assessment (EUnetHTA) has developed a toolkit [18] to support HTA agencies in adapting HTA reports from other countries, regions or settings for their specific use. One of its limitations is that it does not manage the adaptation of HTA reports that are considered primary research. This tool was developed as part of the EUnetHTA HTA Adaptation Toolkit, and focuses on three elements for adaptation: relevance, reliability and transferability. The transferability domain consists of three questions:

(i) How generalizable and relevant are the results and validity of the data and model to the relevant jurisdictions and populations?
(ii) Are there any differences in the following parameters: perspective, preferences, relative costs, indirect costs, discount rate, technological context, personnel characteristics, epidemiological context, factors that influence incidence and prevalence, demographic context, life expectancy, reproduction, pre- and post-intervention care, integration of technology into the healthcare system and incentives?
(iii) Does the evaluation violate the national guidelines for cost effectiveness analysis? The tool is a qualitative instrument and no quantitative score for transferability is produced.

### Critical factors for transferring cost in economic evaluation of health technologies

Several factors may vary between jurisdictions, affecting results of an EE, and preventing transferability of results. For example, Sculpher et al. [5] review shows that four groups are generally retained as the area of variability: the characteristics of the patients, the clinical parameters, the healthcare systems, and the socio-economic aspects. However, the main factor most frequently cited in the literature as generating variability in economic results between locations is the unit costs associated with particular resources, e.g. the absolute or relative prices of resources [5, 19].

Welte et al. [20] grouped 14 factors in three large categories: population, healthcare system and methodological characteristic (Table 1). Eight of these factors affect both costs and results, and 5 exclusively affect the costs of the economic evaluation (three at direct costs and two at indirect costs).

**Table 1:** Transferability factors identified from Welte et al. [20]

Categories	Transferability factors	Direct influence on
Methodological characteristics	Perspective; discount rate; medical cost approach (charges, fees, prices); Productivity cost approach (friction cost method, human capital approach, QALYs)	Costs and effects Costs and effects Direct medical cost Productivity cost
Healthcare system characteristics	Absolute and relative prices in healthcare Practice variation (staff characteristics, characteristics and learning effects of physicians; nurses and hospitals; liability of physicians; type of healthcare facility; organizational characteristics Technology availability (range of licensed products; availability of generics; competition; market form of suppliers; payment of suppliers; incentives to suppliers; supplier-induced demand; healthcare delivery structure; waiting lists; referral patterns; healthcare before and after intervention; quality of care; capacity utilization; economies of scale	Direct medical cost Costs and effects Direct costs
Population characteristics	Disease incidence/prevalence Case-mix (age; sex; race; education; socioeconomic; disease severity; co-morbidity; medical history; concurrent medications; susceptibility) Life expectancy (progression of disease; natural history of the disease; lifestyle; risk factors; environmental factors; genetic factors) Health-status preferences factors (methods to measure health-status valuation) Acceptance, compliance, incentives to patients (technology acceptance; compliance; incentives to patients; insurance level; co-payments; moral hazard) Productivity and work-loss time (friction time; income level and distribution) Disease spread patients (population density; immigration; emigration; travelling; ethical standards)	Costs and effects Costs and effects Costs and effects Effects Costs and effects Productivity cost Costs and effects

Source: Welte et al. [20]

Moreover, Goeree et al. [21] identified, from a literature review, a total of 77 factors that can potentially affect the transferability of EE, grouped into five categories: patient characteristics, disease characteristics, provider, healthcare system and methodology used in the analysis. This classification model was mainly based on the categories described by Welte et al. [20]. The factors related to healthcare system are possibly the most influential on costs. These factors refer to differences in clinical practice, guidelines, or norms across countries. Also, differences in unit prices across jurisdictions, absolute and relative unit costs, the types and magnitude of resources, programs, or services that are available, or the availability of treatment.

Countries also differ in the inputs mix used for healthcare delivery, the organization and structure of the healthcare system, the level of technological innovation, and local technical efficiency in care production.

Some studies [19, 22–30] have addressed the cost transferability of economic evaluation analyses from the original setting to a target country by adjusting the unit costs. Most of them highlight the difficulty of the intended adjustment of cost data due to the poor quality and lack of transparence of the results reported.

Brigitte [24] analyses the adaptation of a cost–effectiveness study of trastuzumab for the adjuvant treatment of HER2-positive early breast cancer from the UK to The Netherlands. They concluded that the model is transferable to The Netherlands, but is still a challenge the availability of health care resource data. More attention should be given to reliable registration of resource consumption related to different health states of diseases like, for example, breast cancer.

Fukuda et al. [25] establish a methodology to deal with cost transferability in economic evaluation. The authors describe four levels of transparency in the reporting of items included in the estimations of cost:

- Level A: all components of costs were described and data for both quantity and unit price of resources were reported for each component;
- Level B: all components of costs were described and data for costs in each component were reported. This included studies that used graphical presentations of the aforementioned data;
- Level C: all components of costs were described, but data for costs in each component were not reported;
- Level D: only the scope of costing was described, but the components of costs were not described.

For example, studies that only reported terms such as ‘‘hospital stay’’ or ‘‘direct costs’’ without further exposition were evaluated at Level D. Additionally, the methodology used to calculate unit cost was also taken into account, categorized according to quality criteria in: (1) micro-costing or quasi micro-costing, (2) use of relative values units, (3) use of ratio of cost to charges, (4) unmodified charge data, and (5) unknown. Finally, the authors also assessed the post-publication number of citations per year for each paper categorized by these evaluation axes of transferability and they found that only 8 out of 79 papers scored a high level of transferability in costs. The most frequent method to estimate cost was the use of charges as proxy of cost, and there was no significant difference in citation frequency between studies with high transferability and low transferability.

Fukuda et al. [25] above methodology for evaluating cost transferability was used by Zwolsman et al. [26]. Authors sought to provide an overview of process cost estimates variability for stress urinary incontinence, exploring factors behind this variation. Results included high heterogeneity in reporting cost estimates, and high variability, underpinned by differences in service provision amongst countries, sources used to derive costs, and the way in which units were defined. Similarly, Ruggeri et al. [27], Gorry et al. [19] and Mandrik et al. [28] highlighted poor costs reporting that makes transferability difficult. Moreover, Ruggery et al. [27] insists on standardizing procedures and having official, independent sources of information for the conduction of economic evaluations.

Steuten et al. [29] provide an overview of critical factors that affect the transferability of economic evaluation in medical devices, and describe the results from a decision-analytic model, developed to assess the cost implications of the use of a fibrin sealant in orthopedic surgery in the UK, were successfully remodeled for France, Germany and Italy.

Authors concluded that economic modeling methods can help transfer data across countries, but empirical research is needed to determine the relative impact of various transferability factors, and impact variation by type of disease, intervention or geographic location.

Finally, Gao et al. [30] explores the transferability of direct medical cost data across countries for some procedures such as schizophrenia, epilepsy and type 2 diabetes mellitus. The authors found that converting raw data into percentage of GDP/per capita of the corresponding individual country could be a feasible approach to transfer the direct medical cost across countries.

### What do national economic evaluation guidelines say about cost transferability?

Barbieri et al. [3] assessed the positions of national pharmacoeconomic guidelines on the transferability (or lack of transferability) of clinical and economic data (key data relating to baseline risk, treatment effect, health state utilities, resource use, and unit costs) and to review guidelines methodology for addressing issues of transferability. They found most guidelines recommend presenting quantities of resources-use separately from unit costs in order to increase transparency. However, 6 out of 22 guidelines did not provide any explicit information on the degree of transferability of resource use. The majority of the remaining guidelines recommend obtaining resource use from the local setting, arguing that estimates from elsewhere have questionable transferability. Differences in clinical practices, payment systems, incentives, and the opportunity to redeploy resources are often mentioned as main reasons for variability in resource use between settings. Guidelines suggest using local data for resource consumption; estimates obtained from other locations are often not considered an appropriate and valid source. However, small countries appear to be more flexible in accepting key data from other settings and, in six cases, estimates of resource use are seen as highly transferable. According to the author, flexibility in accepting data from other jurisdictions would depend on the year of publication, and on the level of methodological development of the guidelines. In addition, some guidelines provide sources for unit costs (e.g., an official list).

Van Dongen [31] reviewed which recommendations are currently given by national pharmacoeconomic guidelines on the statistical analysis of trial-based economic evaluations. The majority of guidelines did not provide recommendations on how to deal with baseline imbalances, skewed costs, correlated costs and effects, the clustering of data, the longitudinal nature of data, and missing data in trial-based economic evaluations.

A revision of methodological guidelines in HEE shows that some guidelines explicitly mention the issue of transferability. For instance, the Austrian Guidelines [32, 33] state that the adaptation of studies can take place in different ways and levels, ranging from relatively simple methods to account for inflation and currency adjustment, to the substitution of data on resources or costs, or even of whole model structures. Indeed, feasibility of transferring data for decision-making in Austria would be conditioned by data availability and level of detail, and the effort invested in adaptation. For instance, if the original studies present only total costs, but not quantity of resources and individual (unit) costs separately, it would be impossible to adapt costs to the Austrian setting. Moreover, guidelines suggest converting prices by means of the use of purchasing power parities (PPP), recommended for currency conversion, since exchange rates lead to distorted results. If both inflation and currency adjustments are carried out, consistency should in any case be the same reference system for purchasing power parities and price indices (e.g. GDP deflator price index and GDP-PPP). The adaptation process is precisely defined. It starts with the currency adjustment, followed by the inflation adjustment. Step three is the adaptation of the discount rate, followed by the conversion of the program costs as well as the cost savings and productivity losses.

The Irish Guidelines indicate that when costs are applied from other countries, the assumptions necessary to transfer this data must be explicitly reported, with all costs converted to their Irish equivalent in euro using Purchasing Power Parity indices. If transferring costs from another country, the inflation should be calculated using the Consumer Price Index for the local currency prior to conversion to the Irish equivalent in Euro using Purchasing Power Parity indexes.

The German Guidelines [34, 35] address the main problems of transferability via Welte et al. [20], and recommends modeling adjustments when there are large differences between study and target country in (1) incidence/prevalence, (2) practice variation, or (3) relative prices. Adjustments may concern the structure of the decision model (to adapt to different health care processes) or the resource utilization. Adjustments of valuation (unit prices) should always be carried out. Furthermore, adjustments should be made for inflation and different currencies. For currency conversion, purchasing power parities are recommended.

The French guideline [36] also mention that an economic evaluation is rarely generalizable to a different context from the one in which it was conducted, and states that although the use of an economic evaluation in another context can however be considered if the interventions being compared are relevant and if the methodology of the study is of good quality, adjustments to the structure or the parameters are always necessary, because of the specific characteristics of the population (incidence/prevalence, life expectancy, preferences, etc.), the healthcare system (organization, professional practices, unit costs, etc.) or methods (time horizons, perspective, discount rates, etc.) which can lead to differences in the evaluation of the costs or health effects. Economic evaluations can be transferred to another context using these adjustments only under certain conditions. The evaluation of the degree of transferability of studies can be used to select studies that meet the necessary explanatory and transparency conditions. Stil, the task of transferring a study is complex and it is necessary to have the full report containing details of all the work and to contact the authors to discuss the conditions for the internal and external validity of their model. Finally, whether transferring a model developed in another context or constructing a model from scratch, the use of foreign data to rate a model's parameters is often unavoidable. The degree of acceptability of foreign data varies according to the nature of the parameter for which information is provided. A distinction can thus be made between the following: (i) variables for which French data are essential (e.g.: calculating the costs of interventions); (ii) variables for which French data are preferable, while accepting the use of foreign data under certain conditions (e.g.: evaluation of quality of life, compliance); and (iii) variables for which foreign data are generally accepted (e.g.: evaluation of the relative risks). The author of the evaluation justifies the balance struck between the value of using foreign data and their validity for a French evaluation.”

The Spanish Guidelines [37] also addresses the issue of transferability and recommend maximum transparency in reporting in order to help decision-makers in generalizing and transferring the various components of an EE to a different setting from the original ones they were intended for. It is strongly recommended that when a study is carried out the authors should be aware that someone else might be willing to use the same study in the future to take decisions in a different setting. It would be convenient to keep this in mind and in order to facilitate the adaptation of the original studies with the minimum amount of additional effort. Generalizability and transferability can be also enhanced by carrying out sensitivity analyses on the appropriate parameters.

### Health care unit cost database and standard cost list

A number of countries have developed standard cost lists to help standardize their economic evaluations. The first countries that applied HEE analysis in the early 1990’s to inform reimbursement decisions of pharmaceuticals and other health technologies were Australia and Canada. Australia and Canada were also the first countries to establish Standard Costs Lists [13, 38]. Australia developed a single national list, whereas Canada issued several provincial lists. In the EU, only three countries have a Standard Costs Lists, or similar documents: the UK, the Netherlands and Germany. The WHO-CHOICE program also developed some unit cost information to support CEA mainly aimed at identifying priorities and to estimate the global cost of publicly financed health benefits packages for middle and low-income countries [39]. Table 2 provides a summary of main cost databases published for HEE.

**Table 2:** List of the main current documents related to costing and standard unit costing in healthcare

Country	Cost methodology manual/cost list/database cost for Hee	Year
Australia	Manual of resource items and their associated unit costs. For use in submissions to the Pharmaceutical Benefits Advisory Committee involving economic analyses Version 5.0 [40]	December, 2016
Canada	Guidance Document for the Costing of Health Care Resources in the Canadian Setting Second Edition [42]	June 2015
	Canadian Patient Cost Database Technical Document MIS Patient Costing Methodology^a^	January 2019
United Kingdom	Unit Costs of Health and Social Care. Complete document [43]	2018
	Unit cost database of health and social care professionals^b^	2017/18
Netherland	Update of the Dutch Manual for Costing in Economic Evaluations. [44]	2017
Germany	Working Paper on Cost Estimation in health economic evaluations [34]	2009
Thailand	Standard cost lists for health economic evaluation in Thailand [48]	2014

Source: own elaboration

^a^Available at: https://www.cihi.ca/sites/default/files/document/mis_patient_cost_meth_en_0.pdf

^b^Available at: https://www.pssru.ac.uk/project-pages/unit-costs/

In Australia, from 1993 onwards, the Commonwealth Department of Health, Housing and Community Services (DHHCS) requires submissions to the Pharmaceutical Benefits Advisory Committee (PBAC) for the listing of a new Drug on the Pharmaceuticals Benefits Scheme (PBS) to incorporate a clinical as well as an economic evaluation, which must follow the available guidelines. In August 1992 a Manual of Resource Items and their Associated Costs for use in submissions to the PBAC involving economic analyses was developed as a complement of the Guidelines, in order to ensure consistency and comparability. The Manual included definitions and descriptions and some methodological recommendations, as well as several sub-lists of resource items with their monetary values attached. The Manual has been updated several times. The most recent Version 5.0 was published in 2016 [40]. This version does not include an explicit list of resource items with their respective unit values, but it redirects the user to the originals sources by means of hyperlinks to the various categories of resources.

In Canada, the first document with specific guidance for costing appeared in 1996—Canadian Coordinating Office for Health Technology Assessment [41]. This guidance was updated in 2015 [42]. The present version contains only detailed information, and methodological advice, as well as links to the adequate sources of cost information at provincial level, a normal feature in a federal state.

In the United Kingdom, the reference source for unit costs for economic evaluation in health and social care is a project led by the Personal and Social Services Research Unit (PSSRU) of the University of Kent that started in 1992. It is basically funded by the Department of Health and Social Care, with a minor contribution from the Department of Education. The flagship piece of work of the project is the annual report. The most recent issue is Unit Costs of Health and Social Care 2018 [43], available at https://www.pssru.ac.uk/project-pages/unit-costs/unit-costs-2018/. Annual reports can be downloaded as pdf from the website since 2003. Its main function is to generate a source of unit costs. Unit cost is defined as the cost of an unit of output and the basic approach is to estimate it as long-run marginal opportunity cost, based on existing studies and on new studies that carry out internally or that they commission to external researchers. They work in close collaboration to the NICE, among other institutions. The project has a website and has recently developed a repository of studies among other web-based resources, such as data spreadsheets.

In Netherland, the first “Dutch Manual for Costing: Methods and Reference Prices for Economic Evaluations in Healthcare” was published in 2002, followed by an updated version in 2004 and 2010 [44]. The purpose of the Manual is to facilitate the implementation of costing studies in economic evaluations. Kanters et al. [45] describes the update of the Dutch costing manual. The updated costing manual is freely available from the website from ZIN (www.zorginstituutnederland.nl) together with an online Microsoft Excel instrument containing all updated reference prices and parameters in the costing manual to ensure accessibility (available through www.imta.nl/costingtool). The Dutch Manual is very comprehensive and recommendations are based on theoretical reasons, but it also has a practical approach, as it provides standard costs that can easily be applied to economic evaluations.

In Germany the document of reference regarding costing methodology for economic evaluation in health care is the Institute for Quality and Efficiency in Health Care (IQWiG) Working Paper Cost Estimation Version 1.0—19/11/2009 [35]. Although the IQWiG Working Paper does not provide unit costs, it refers readers to the consensus proposal: “Methods in Health Economic Evaluation” Working Group of the German Society for Social Medicine and Prevention (AG Methoden der Gesundheitsökonomischen Evaluation, AG MEG) [46] which addresses a set of methodological issues on costing health care resources and provides as well a set of standard costs figures for the most relevant health services and resources (82 items).

Thailand, is one of the few middle-income countries for which there is evidence that it has developed a Standard Costs List for HEE. HEE is a standard tool regularly used in Thailand for decisions regarding the inclusion of a new drug on the national list of essential drugs and a new treatment regimen on the national health insurance benefit package. In 2008 the Health Intervention and Technology Assessment Program (HITAP) of the Ministry of Public Health developed National HTA Guidelines, [47] which included a chapter on measurement of costs (Standard cost lists for health economic evaluation in Thailand) also published as a journal article, [48]. A very detailed description and justification of the process, methodology and results of the project is provided in the article from Riewpaiboon 2014 [49].

### Usefulness of an European health care and social costs database

An European health care and social costs database is likely to have several positive effects on the cross-border collaboration and on the efficiency of HEE in the EU. The main purpose of the European health care and social costs database is to develop a tool facilitate the elaboration of high-quality, multi-country economic evaluation analyses of health interventions and programs, and ensure appropriate transference of EE analyses originally carried out in a given EU Member State to other EU members. This cost database could promotes the standardization of health care resources concepts and terms in all countries, and to provide a repository of monetary values for each resource. It also will address two challenges of EE: transferability and transparency. It will improve transferability by making it easier to carry out multi-country studies and to adapt economic evaluation studies from country to country, hence saving time in the task of looking for healthcare and social costs. International standardization of the methodology across jurisdictions is one way to make HEE comparable and to ensure transparency in the decision making among jurisdictions [50].

The core of the database contains three main categories of resources (primary resources, composite goods and services, and complex processes and interventions) further organised into subcategories (Table 3). ‘Primary resources’, those that cannot be broken down into smaller units, can be defined in a standard way by their name or dose (medicines) or manufacturer’s name and model (devices or medical devices). However, other resources such as the cost of the healthcare professional have more variability between countries due to the activity that is included in their cost/hour, the methodology for allocating the activity in the cost item, as well as the type of activity that is performed, or who performs that activity. ‘Composite goods and services’ would encompass several primary resources that are consumed together. For example, a day in hospital will include some staff activity (nursing, doctors' rounds), some equipment services (catering, laundry) and often overheads (energy, general maintenance, porterage, etc.). The cost database should capture whether there are major differences in the definition of these services between countries. There may be very similar items, but with different resource consumption depending for example on where the activity is performed (primary, hospital, outpatient) or professionals performing the activity. ‘Complex processes and interventions are units of activity that aggregate various types of resources (such as procedures, length of stay and primary resources). Many countries use a DRG system to classify hospital admissions or discharges.

**Table 3:** Categories and subcategories within which costing items are organized in the European Healthcare and Social Cost Database

Category	Subcategory
Primary (homogenous) resources	Medicines
	Medical devices
	Health products/disposables
	Personnel
Composite goods and services	Outpatient visit
	Hospitalization
	Image diagnosis
	Laboratory tests
	Ambulance service
	Diagnostic procedures
	Therapeutic procedures
Complex processes and interventions	Inpatient medical and surgical processes
	Day case procedures/outpatient surgery

Source: own elaboration

Ensuring comparability requires detailed information on costing methodologies used to estimate cost of each item included in the EU HCSCD as possible. This means the knowledge about what resources are included in the cost, how were the resources estimated and how was the unit cost calculated and assigned to the resources. A free-access Beta version of the database is available at https://www.easp.es/Impact-Hta/default15 together with the User’s manual and all documents/papers describing some aspects of costing methodology published so far. In the long term, comparability of costs in HEE could be easily attained if all countries/institutions used the same accounting methodology. In the meantime, it would make sense to promote the joint work and collaboration of HEE analysts and users, by building appropriate tools, such as the European Health Care Costs Database (EU HCCD), that allows transference and adaptation of studies in the EU. In order to ensure the continuity of the EU HCCD an open, collaborative project should be designed that institutionalises the initiative beyond the end of the HAT Impact project.

The EU HCCD includes unit costs that mainly refer to countries/nations, but might be extended to costs representing regions or smaller areas and settings, if they are relevant jurisdictions for decision making and unit cost data are available and can be collected. If there are within-country regions or jurisdictions characterized by large differences in costs, assessing the cost-effectiveness of a decision should ideally be based on the differential costs at each region/jurisdiction/setting. That is, an intervention that reduces length of hospital stay after a surgical procedure, might be cost effective for a tertiary hospital with a high cost per day but not cost-effective for a local hospital with much lower hospitalization costs.

## Conclusions

Variation in unit cost estimates of a given item between countries can arise for several reasons, among them: differences in the definition of the unit of activity, differences in the quantity, quality or mix of resource inputs used in providing a service or activity, differences in average price levels across countries, differences associated with the currency exchange rate, or differences in the cost-accounting methodology applied to estimate the cost item.

EE is an important analytical tool to inform and facilitate an efficient decision-making for resource allocation in the health sector, mainly in funding and pricing technologies. However, EE are costly, time-consuming exercises. Variation in methodological requirements and decision-making criteria by jurisdictions are recognized barriers to the generalizability of HEE across jurisdictions, making transferability more difficult and time-consuming but not preventing it. Several revisions have highlighted the poor quality and lack of transparency of cost data reported in EE [19, 25, 26]. Moreover, few jurisdictions’ HEE guidelines contain detailed procedures and methodologies for costing resources. It is even rarer for jurisdictions to provide explicit lists of single standard unit cost per resource item, an option that would greatly facilitate calculations and prevent intentional biases in calculating unit costs.

The European Healthcare and Social Cost Database (EU HCSCD) for use in Health Technology Assessment (HTA) across countries has been developed to facilitate the elaboration of high-quality, multi-country economic evaluation and ensure appropriate transference of EE analyses originally carried out in a given EU Member State (EUMS) to other EUMS. This cost database promotes the international standardization of health care resources concepts and terms in all countries, transferability and transparency.

## Supplementary Information

**Additional file 1:** **Table S1**. Search strategy for Medline and Web of Knowledge.

## Data Availability

Not applicable.

## Abbreviations

WP3: Work Pakage 3
EU HCSCD: European health care and social costs database
LR: Literature review
HTA: Health technology assessment
HEE: Health economic evaluation
P&R: Pricing and reimbursement
EUnetHTA: European Network for Health Technology Assessment
