# Supplementary material for: Cost transferability problems in economic evaluation as a framework for an European health care and social costs database
Source: Cost Eff Resour Alloc. 2021 Jul 18;19:43. doi: 10.1186/s12962-021-00294-4 (PMC8286608; doi:10.1186/s12962-021-00294-4)
Supplement: Supplementary file 1 — Additional file 1: Table S1. Search strategy for Medline and Web of Knowledge. [file 12962_2021_294_MOESM1_ESM.docx]

Additional file 1

Table S1. Search strategy for Medline and Web of Knowledge

**Search strategy for Medline**

|  |  |  |
| --- | --- | --- |
| Search | *Terminology used for Medline and Web of Knowledge* | **Result** |
| *Medline*  *Web of Knowledge* | *"Health Care Costs"[Mesh]) AND ("standard cost list" or "database" or "transferability" or "toolkit" or "cost database" or "guideline" or "check list") and ("economic evaluation" or "Cost-Benefit Analysis"[Mesh]) Filters: in the last 15 years, English, Spanish*  *(cost) AND ("standard cost list" or "database" or "transferability" or "toolkit" or "cost database" or "guideline" or "check list")and (("economic evaluation" and ("health technology" or "medicine" or "pharmaceutical")))* | 640  513 |
